# Participation in One Health Networks and Involvement in the COVID-19 Pandemic Response: A Global Study

**DOI:** 10.3389/fpubh.2022.830893

**Published:** 2022-02-24

**Authors:** Laura C. Streichert, Ludovico P. Sepe, Pikka Jokelainen, Cheryl M. Stroud, John Berezowski, Víctor J. Del Rio Vilas

**Affiliations:** ^1^One Health Commission, Apex, NC, United States; ^2^Department Biological Safety, German Federal Institute for Risk Assessment (BfR), Berlin, Germany; ^3^Infectious Disease Preparedness, Statens Serum Institut, Copenhagen, Denmark; ^4^Scotland's Rural College, Inverness, United Kingdom; ^5^World Health Organization, Regional Office for South-East Asia, New Delhi, India

**Keywords:** SARS-CoV-2, One Health, network, multisectoral, pandemic response, capacity-building

## Abstract

The COVID-19 pandemic exemplifies a One Health issue at the intersection of human, animal, and environmental health that requires collaboration across sectors to manage it successfully. The global One Health community includes professionals working in many different fields including human medicine, veterinary medicine, public health, ecosystem health, and, increasingly, social sciences. The aims of this cross-sectional study were to describe the involvement of the global One Health community in COVID-19 pandemic response activities. One Health networks (OHNs) have formed globally to serve professionals with common interests in collaborative approaches. We assessed the potential association between being part of an OHN and involvement in COVID-19 response activities. Data were collected in July-August 2020 using an online questionnaire that addressed work characteristics, perceived connection to OHNs, involvement in COVID-19 pandemic response activities, and barriers and facilitators to the involvement. The sample included 1,050 respondents from 94 countries across a range of organizations and work sectors including, but not restricted to, those typically associated with a One Health approach. Sixty-four percent of survey respondents indicated involvement in pandemic response activities. Being part of an OHN was positively associated with being involved in the COVID-19 response (odds ratio: 1.8, 95% confidence interval: 1.3–2.4). Lack of opportunities was a commonly reported barrier to involvement globally, with lack of funding the largest barrier in the WHO African region. This insight into diverse workforce involvement in the pandemic helps fill a gap in the global health workforce and public health education literature. An expanded understanding of the perceived roles and value of OHNs can inform targeted interventions to improve public health education and workforce capacity to prepare for and respond to public health emergencies.

## Introduction

The COVID-19 pandemic is a complex issue that has affected almost every aspect of life worldwide (1, 2). It has led to the mobilization of a public health workforce and to community engagement campaigns in diverse contexts around the globe. Managing the pandemic requires strategies that facilitate communication and coordinate action across sectors and disciplines. One Health is an operational framework that takes an integrated, multisectoral, and transdisciplinary perspective, with a focus on the links between animal, human, and environmental health systems (3). The COVID-19 pandemic is considered a One Health issue because of its complexity and the zoonotic nature of the coronavirus SARS-CoV-2 (4–6).

The need for a coordinated One Health approach to mitigate and address pandemic risks, including COVID-19, has been embraced by leading international policy organizations, including the Tripartite made up of the World Health Organization (WHO), the World Organisation for Animal Health (OIE), and the Food and Agriculture Organization of the United Nations (FAO) (7); the United Nations Environment Programme (UNEP) (8); the World Bank (9–11); and others (12–14). The release of a working definition for One Health with joint Tripartite and UNEP support demonstrates the momentum for operationalizing coordinated One Health approaches at multiple levels in the international arena (3). While there has been extensive rhetoric supporting the One Health concept and approach during the current pandemic, the impact of One Health networks on the extent of multisectoral workforce response to the COVID-19 pandemic has not been investigated on a global scale.

The proof of concept for the utility of One Health has been demonstrated repeatedly during previous outbreaks of zoonotic diseases (13, 15–17). The key messages from One Health actions reported during COVID-19 include the importance of a supportive environment with shared resources, interdisciplinary engagement, and strategies for communication networks (18, 19). To perform effectively, professionals need to be armed with the knowledge and skills from their own discipline, and also to be motivated and able to bridge with others (20). Proficiency in competencies required for understanding and applying One Health concepts requires breaking down disciplinary and professional siloes to find areas of overlap and complementarity (21–24).

Worldwide, One Health networks (OHNs) play a role in operationalizing One Health by providing information sharing, professional development, and opportunities for collaboration across disciplines (25–27). In April 2020, the WHO Global Outreach and Response Network (GOARN), in partnership with the One Health Commission (OHC) and the One Health European Joint Programme (One Health EJP), issued a COVID-19 Call to Action seeking experts in One Health to assist during the pandemic (28). The rapid response to the call from over 600 professionals working in anthropology, medicine, epidemiology, veterinary care, wildlife, public health, ecohealth, and other disciplines demonstrated the potential of OHNs to reach and mobilize a diverse workforce. It also highlighted the need for more research to evaluate the outcomes and impacts of OHNs.

While the application of One Health approaches has been evaluated in a number of contexts (29–31), there has been relatively little assessment of the impact of OHNs or factors that support workforce efforts to operationalize One Health (19, 32–34). The aims of this study were to describe the involvement of a cross-section of the global One Health community in the COVID-19 pandemic response, to discern the barriers and facilitators that influenced that involvement, and to elucidate any connection between being associated with an OHN and involvement in COVID-19 response activities. This is the first study to examine the reach and impact of OHNs as determined by primary data across multiple contexts and, therefore, has relevance for many different audiences, including the general public.

## Materials and Methods

### Study Design and Recruitment

We conducted a questionnaire-based descriptive study and report the work and its results following the STROBE checklist for cross-sectional studies (35) (Supplementary Material 1). The questionnaire was administered using an online survey tool (Survey Monkey), with no restrictions to respondents. The survey link was distributed broadly through OHN listservs, social media, and to over 100 previously identified OHNs (25), with a request to distribute it beyond the OHNs. The survey link was open from 15 July 2020 to 21 August 2020.

### Questionnaire

The English-only questionnaire (Supplementary Material 2) was piloted with a group of individuals from different sectors (human medicine, public health, animal health) and types of organizations (academic, non-profit), and revised to ensure clarity and consistency before its launch. The questions and response options were developed based on previous work on the topic (25) and the experience of the diverse, multidisciplinary project team (20). A definition for One Health was provided in the introduction of the survey instrument. The sixteen questions covered selected key work-related characteristics of the respondent; self-reported connection with an OHN and participation in OHN activities; self-reported involvement in COVID-19 response; skills applied and activities conducted as part of the pandemic response, if applicable; and perceived barriers and facilitators to involvement in the COVID-19 response.

As OHNs include both formal and informal structures, and in an effort toward inclusion, a definition for an OHN was not provided in this study. For purposes of this study, the sense of connection to an OHN by the survey respondent was of greatest importance, which we believed should not have been constrained by a definition.

The COVID-19 response was defined as response and/or research related to the pandemic. For categorizing the respondents by geographic regions, the WHO list of member countries and regions was applied (www.who.int/countries). For several questions, such as those concerning the type of organization and sector, the survey respondents were able to select multiple options from the list of possible answers, including the opportunity to select “other.” Those survey respondents who were involved in a response were asked to indicate the type of work, geographic level of response, and skills and areas of expertise applied.

### Statistical Analyses

We describe the data according to the background variables captured by the questionnaire. Since not all respondents answered all questions, we report the total number of respondents (N) who answered each question.

The main results are simple distributions, presented as counts and percentages. Differences between relevant proportions were evaluated using the Chi-square test, and considered statistically significant if the 2-sided *p*-value was *p* < 0.05. The sample size we aimed for was targeted for general descriptive statistics, and subgroup analyses were not a main objective.

We report odds ratio (OR) from a logistic regression model where the outcome was the reported involvement in COVID-19 response (yes/no), and dichotomous: “being part of an OHN” (yes/no) was the explanatory variable. We additionally evaluated the association with the sectors represented by at least 400 responses to the question (dichotomous variable: selected/not selected). Confounding was explored by observing any substantial change in OR after adding each of the variables, and interaction was tested for by offering an interaction term to the model. The predictive power of the logistic regression models is presented as the area under the receiver operating characteristic (ROC) curve.

Statistical tests were performed using GraphPad Prism version 6.0.1 for Windows (GraphPad Software, San Diego, California, USA; www.graphpad.com) and Stata 13.1 (StataCorp, College Station, TX, USA).

### Ethics Approval

The research was exempted from ERC review by the Ad-Hoc Covid-19 Research Ethics Review Committee (WHO ERC/Covid-19). A link to a Participant Information Sheet (PIS) was included in the survey instructions (Supplementary Material 3).

Participation was completely voluntary, no questions were mandatory to answer, and the respondents consented for their answers being used by submitting them. The data were anonymous; the dataset was checked for completeness of anonymity and de-identified (Supplementary Material 4). No potentially identifiable human data are presented in this study.

## Results

### Subject Population

**Figure 1:**
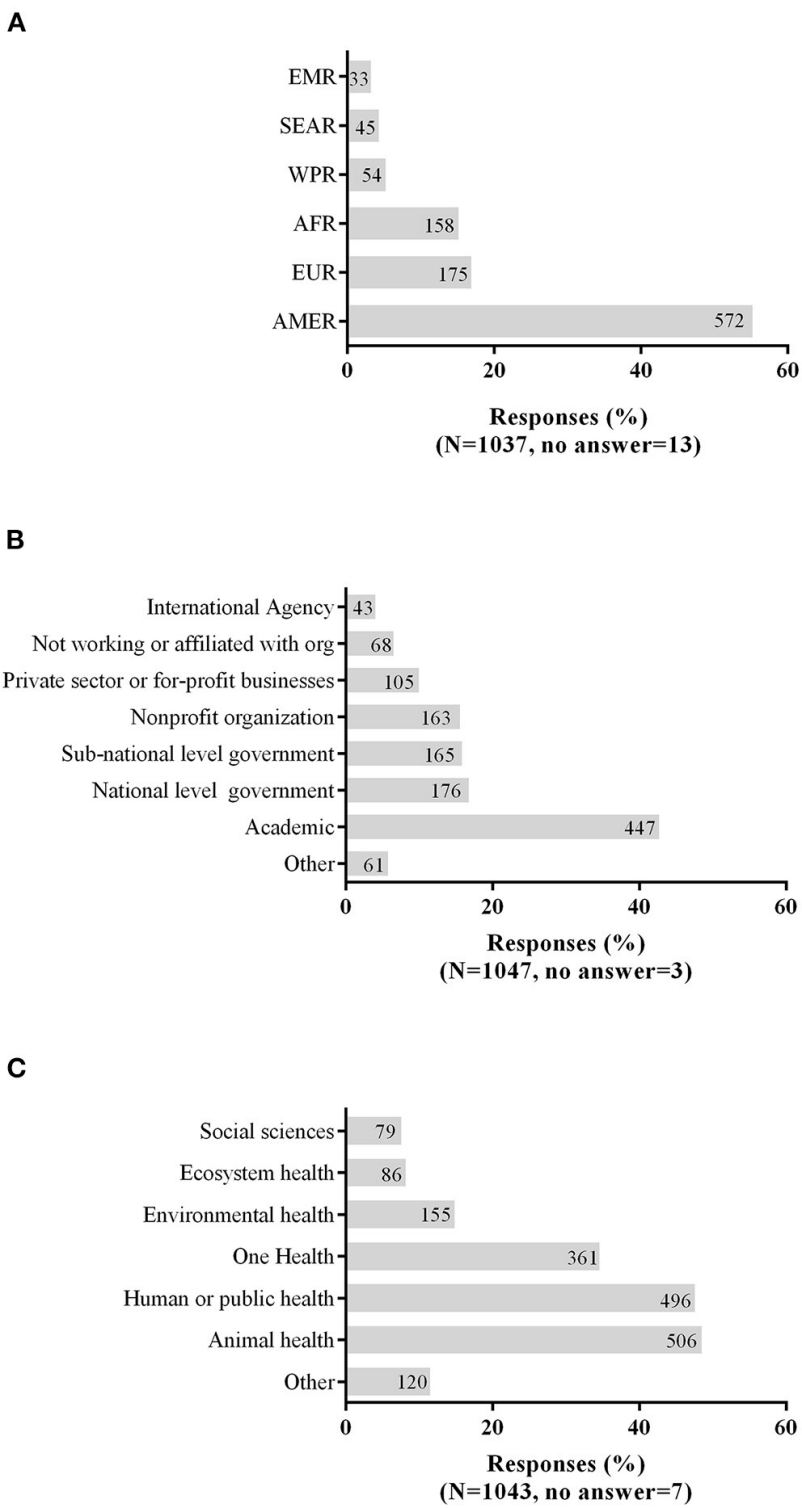
Survey respondents by **(A)** WHO region, **(B)** type of organization, and **(C)** work sector.

The sample for this observational study included 1,050 respondents who were categorized by three relevant work variables—location (WHO region), type of organization, and work sector (Figure 1). The respondents were from 94 countries in all six WHO regions (Figure 1A). A large proportion of survey respondents who answered the question were from the Region of the Americas (572/1,037, 55.2%); 44.6% (462/1,037) were from the United States. Academic organizations (Figure 1B) were the most commonly selected affiliation (447/1,047, 42.7%), followed by governmental organizations at the national (176/1,047, 16.8%) and sub-national (165/1,047, 15.8%) levels.

The survey respondents were from a variety of work sectors (Figure 1C). A similar percentage reported working in animal health (506/1,043, 48.5%) and in human health or public health (496/1,043, 47.6%). The environmental health sector was selected by 14.9% (155/1,043) of respondents. Over one-third of respondents (361/1,043, 34.6%) self-identified as working in the One Health sector.

Overall, 13.2% (138/1,047) of the survey respondents indicated that they were working for or were affiliated with more than one type of organization (Supplementary Table 1). One Health sector respondents were more likely to report working for more than two types of organization (*p*-value: < 0.001); a total of 20.8% (75/361) of those self-identifying as working in the One Health sector reported working for more than one type of organization, compared to 9.2% (63/686) among those working in all other sectors combined.

The majority (299/361, 82.8%) of respondents who reported working in One Health sector also selected at least one other sector; 50.7% (183/361) selected at least two additional sectors. This is a higher proportion compared to 18.0% (123/682) who reported working in more than one sector and 4.4% (30/682) who reported working in more than two sectors among those who did not identify as working in the One Health sector (*p* < 0.001; Supplementary Table 1).

### Participation in One Health Networks (OHNs)

Overall, 75.7% (788/1,041) of survey respondents identified as being part of an OHN. The number and percent of survey respondents, categorized by being self-described as part of an OHN, are presented by WHO region, type of organization, and work sector in Table 1. Across all WHO regions, all types of organizations, and all work sectors, the proportion of the sample reporting they were part of any OHN was always above 65.8%. The top three OHN activities that most survey respondents indicated they had ever participated in were, “received communication from an OHN,” “social media communications,” and “OHN- hosted webinars” (Table 2). Supplementary Table 2 provides a summary of the characteristics of those respondents who indicated being part of an OHN compared to those who did not.

**Table 1:** Survey responses for being part of an OHN, involved in COVID-19 response, and both part of OHN and involved in COVID-19 response, by WHO region, type of organization, and work sector.

	**Part of OHN**		**Involved in**		**Part of OHN and**	
			**COVID-19 response**		**involved in COVID-19 response**	
	***n***	**%**	***n***	**%**	***n***	**%**
***Where are you currently located? (WHO Region)***						
Americas (572)	395	69.1	349	61.1	258	45.1
Europe (175)	127	72.6	111	63.4	81	46.2
Africa (158)	142	89.9	110	69.6	104	65.8
Western Pacific (54)	45	83.3	30	55.6	28	51.8
South-East Asia (36)	40	88.9	30	66.7	27	60.0
Eastern Mediterranean (33)	29	87.9	23	69.7	21	63.6
What type of organization do you currently work for or are you affiliated with?^*^						
Academic (447)	349	78.1	282	63.1	226	50.5
National level government (176)	139	79.0	124	70.5	108	61.3
Sub-national level government (165)	123	74.5	124	75.2	94	56.9
Non-profit organization (163)	131	80.4	109	66.9	96	58.8
Private sector or for-profit businesses (105)	74	70.5	69	65.7	36	34.2
Individual not working or affiliated with organization (68)	53	77.9	35	51.5	28	41.1
International Agency (37)	37	86.0	35	81.4	29	67.4
Other (61)	42	68.9	36	59.0	29	47.5
In what sector do you currently work?^*^						
Animal health (506)	402	79.4	279	55.1	227	44.8
Human or public health (496)	383	77.2	381	76.8	302	60.8
One Health (361)	318	88.1	257	71.2	228	63.1
Environmental health (155)	122	78.7	107	69.0	90	58.0
Ecosystem health (86)	76	88.4	59	68.6	55	63.9
Social sciences (79)	60	75.9	60	75.9	50	63.2
Other (120)	79	65.8	69	57.5	0.0	0.0

*N, number of people that answered the question; n, number of responses*

* *Possible to select multiple options, including “other,” from a list. Sum of group percentages does not = 100%*.

**Table 2:** Participation in OHN activities.

	***n***	**%**
Please indicate if you have ever participated in these OHN activities.^*^ **(*N* = 892, no answer = 158)**		
Received communications from OHN list	474	53.1
Followed OHN on social media	379	42.5
Attended OHN hosted webinar	369	41.4
Attended online OHN conference/meeting	332	37.2
Attended in-person OHN conference/meeting	297	33.3
Invited other professionals to OHN activities	251	28.1
Used OHN to disseminate information	200	22.4
Participated in OHN workgroup/taskforce/committee	190	21.3
Participated in integrated OHN project	184	20.6
Organized OHN activity	180	20.2
Co-authored OH publication with OHN colleague	143	16.0
Participated in OHN offered training	141	15.8
Presented on OH topic for OHN	129	14.5

*N, number of people that answered the question; n, number of responses*

* *Possible to select multiple options, including “other,” from a list. Sum of group percentages does not = 100%*.

### Involvement in COVID-19 Response

A total of 63.8% (661/1,036) of survey respondents indicated that they had been involved in COVID-19 response activities. Table 3 summarizes the answers from survey respondents who were involved in the COVID-19 response regarding the type of work, geographic level of response, and skills and areas of expertise applied. The largest percentage of the respondents (309/681, 45.4%) indicated they were involved in education, including teaching and training. Over half the respondents indicated that their response activities were at the subnational level (364/651, 55.9%). The skills and areas of expertise applied included animal health (328/698, 47.0%), disease surveillance (255/698, 36.5%), and information/knowledge management (226/698, 32.4%).

**Table 3:** COVID-19 response actions by type of work and by geographic level of response, and COVID-19 response actions by skills and areas of expertise applied.

	***n***	**%**
What is your type of work for the COVID-19 response?^*^		
(*N* = 681, no answer = 82, did not participate in COVID-19 response = 287)		
Education (teaching, presentation, training)	309	45.4
Practice (clinical, public health, lab support, data analysis)	264	38.8
Writing (blog, commentary, article, other publication)	187	27.5
Health policy and consultation	173	25.4
Research (basic, clinical, operational)	138	20.3
Administration and support	130	19.1
Research (social science, fieldwork)	127	18.6
Research (COVID-19 diagnostics, treatments, or vaccines)	90	13.2
Other	98	14.4
At what level is your COVID-19 response and/or research activities?^*^		
(*N* = 651, no answer = 89, did not participate in COVID-19 response =310)		
Subnational—local, district, state	364	55.9
National—in one country	301	46.2
International—in multiple countries	144	22.1
Other	0.0	0.0
***What skills/areas of expertise have you applied to the COVID-19*** response?^*^		
(*N* = 698, no answer = 81, did not participate in COVID-19 response = 271)		
Animal health	328	47.0
Disease surveillance	255	36.5
Information/knowledge management	226	32.4
Communications and media	220	31.5
Community engagement	180	25.8
Risk assessment and management	172	24.6
Risk communications	162	23.2
Infection and Prevention Control (IPC)	160	22.9
Outbreak or epidemiological research	150	21.5
Data management	136	19.5
Environmental health	123	17.6
Basic research on coronavirus	114	16.3
Laboratory support and diagnostics	112	16
Contact tracing	111	15.9
Social sciences	88	12.6
Logistics/supply chain	68	9.7
Case management	64	9.2
Testing and diagnostics development	56	8.0
Human clinical care	52	7.4
Operational research	51	7.3
Clinical research	50	7.2
Vaccine development	19	2.7
Other	59	8.5

*N, number of people that answered the question; n, number of responses*.

* *Possible to select multiple options, including “other,” from a list. Sum of group percentages does not = 100%*.

Among the survey respondents indicating they were involved in the COVID-19 response, 79.9% (528/661) reported being part of an OHN. Among respondents indicating they were part of an OHN, 67.2% (528/786) were involved in COVID-19 response activities. The proportion involved in the pandemic response was smaller at 53.2% (133/250) among those who did not identify as being part of an OHN. Being part of an OHN was positively and significantly associated with involvement in the pandemic response with a univariable odds ratio of 1.8 (95% confidence interval: 1.348–2.405); the area under the ROC curve was 0.555 (Table 4).

**Table 4:** Contingency table showing the association (odds ratio: 1.8, 95% confidence interval: 1.3–2.4, Chi-square: 16.04) between being part of an OHN and being involved in the COVID-19 response.

	**Involved in**	**Not involved**	**No answer**	**Totals**
	**COVID-19**	**in COVID-19**	**for COVID-19**	
	**response**	**response**	**response**	
**Part of OHN**	528	258	2	788
**Not part of OHN**	133	117	3	253
**No answer for OHN**	2	3	4	9
**Totals**	663	378	9	1,050

Two univariable logistic regression models investigating the association between being from the two most commonly selected sectors and being involved in COVID-19 response activities showed that being from the animal health sector was negatively associated with involvement in the pandemic response, and being from the human health or public health sector was positively associated with involvement in the pandemic response (odds ratio 0.5, 95% confidence interval: 0.382–0.639, and odds ratio 3.1, 95% confidence interval: 2.393–4.091, respectively). Further, two separate models including each of these two sectors as an explanatory variable alongside being part of an OHN as the main focus explanatory variable, supported the results of the univariable analyses, and there were no substantial changes in odds ratios. The first model showed that being from the animal health sector was negatively associated (odds ratio 0.5, 95% confidence interval: 0.353–0.597) while being part of an OHN was positively associated (odds ratio 2.0, 95% confidence interval: 1.481–2.688) with being involved in COVID-19 response; area under the ROC curve was 0.619. The second model showed that being from the human health or public health sector was positively associated (odds ratio 3.1, 95% confidence interval: 2.381–4.094) and being part of an OHN was positively associated (odds ratio 1.8, 95% confidence interval: 1.318–2.406) with being involved in COVID-19 response; area under the ROC curve was 0.663. No interaction was evident between either of the sets of two explanatory variables. The sample size did not allow a similar analysis of the environmental sector.

### Barriers and Facilitators to Involvement in COVID-19 Response

Overall, 38.3% (387/1,011) of survey respondents reported no perceived barriers to their participation in COVID-19 response activities. The most frequently reported barrier was “no financial support” (248/1,011, 24.5%), followed by “lack of opportunity or path for involvement” (211/1,011, 20.9%). Personal and organizational interest were the greatest facilitators for involvement (Table 5).

**Table 5:** Barriers and facilitators to participation in COVID-19 response activities.

	***n***	**%**
Barriers to participation in COVID-19 response^*^(***N*** **=** **1,011, no answer** **=** **39)**		
There were no barriers to my participation	387	38.3
No financial support	248	24.5
No opportunity or path for involvement	211	20.9
Not part of my job	172	17.0
No time	149	14.7
Don't know how to get involved	143	14.1
Lack of organizational interest	94	9.3
Lack of personal interest	24	2.4
Other	123	12.2
Facilitators to participation in COVID-19 response^*^(***N*** **=** **1,015, no answer=** **35, did not participate in COVID-19 response** **=** **295)**		
Personal interest	508	50.1
Organizational interest	391	38.5
I have not participated in COVID-19 response and/or research	295	29.1
Part of established duties at my current job	295	29.1
Part of a new project/special deployment for COVID-19	223	22.0
Availability of new COVID-19 funding	124	12.2
I learned of volunteer opportunity through OHN	42	4.1
I learned of job opportunity through OHN	21	2.1
Other	59	5.8

*N, number of people that answered the question; n, number of responses*.

* *Possible to select multiple options, including “other”, from a list. Sum of group percentages does not = 100%*.

**Figure 2:**
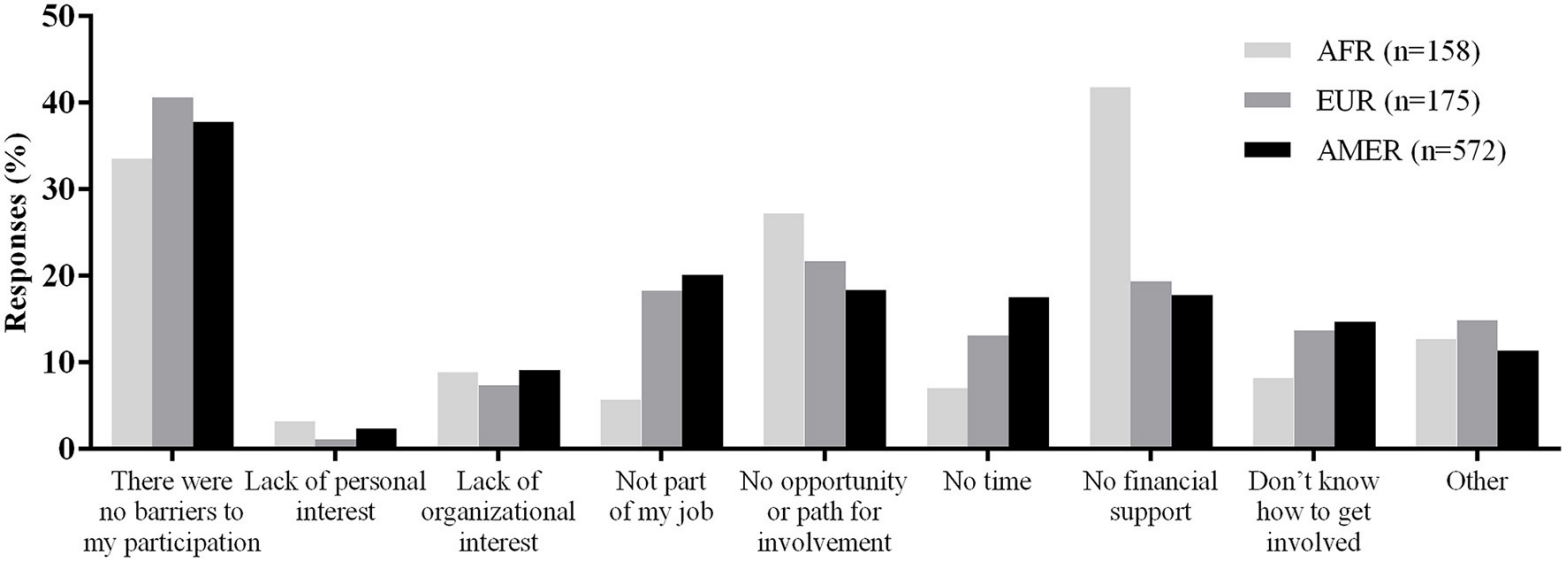
Perceived barriers to participation in COVID-19 response by WHO region.

Figure 2 presents the perceived barriers to involvement in the COVID-19 response for the WHO regions that were represented by at least 100 responses to the question, notably the European, African, and Americas regions. Barriers perceived by survey respondents from the European and Americas regions were generally similar to one another. The most frequently reported barrier by respondents from the African region was “lack of financial support” (66/158, 41.8%). The proportion selecting this as a barrier was significantly lower in the other two regions: 19.4% (34/175) in the European region and 17.8% (102/572) in the Americas (*p* < 0.001). A significantly lower percentage of respondents from the African region indicated not knowing how to get involved (13/158, 8.2%), compared to respondents from the Americas (84/572, 14.7%; *p* < 0.05).

### Perceived Usefulness of OHNs During COVID-19 Response

While 43.6% (235/539) of the survey respondents who affiliated with an OHN that contributed to the COVID-19 response found OHNs very helpful or extremely helpful, 19.1% (103/539) found OHNs to be of little or no help (Supplementary Figure 1). The OHN offerings most frequently reported as especially useful were “increasing public awareness of the value of One Health” (712/923, 77.1%) and “networking with professionals across sectors with common interests” (517/923, 56.0%; Table 6).

**Table 6:** Perceived usefulness of OHN activities during the COVID-19 response.

	***n***	**%**
What OHN offerings do you think are especially useful during the COVID-19 response?^*^		
(*N* = 923, no answer = 127)		
Increased public awareness of the value of OH	712	77.1
Networking with professionals across sectors with common interests	517	56.0
Trusted information about the COVID-19 pandemic	466	50.5
Links to popular media items relevant to OH and current events	338	36.6
Targeted training opportunities	335	36.3
Information about professional, career, and service opportunities	310	33.6
Opportunities to contribute in ways that my employment does not provide	269	29.1
Other	33	3.6

*N, number of people that answered the question; n, number of responses*.

* *Possible to select all that apply from list of options, including “other.” Sum of percentages does not = 100%*.

## Discussion

One Health has been invoked on the international stage as a major principle with which to fight the COVID-19 pandemic and to prevent future pandemics. This study describes the contributions of a cross section of the global One Health community that included a broad representation that spanned geographic regions, organizations, and work sectors in the early stages of the COVID-19 pandemic. It captures what individuals regarded as barriers and facilitators to their involvement, including the role of participating in an OHN, and reveals where further research is warranted.

Being part of an OHN was positively associated with involvement in COVID-19 responses. This provides evidence for the value of OHNs for workforce capacity building. However, not all respondents found OHNs helpful. For those survey participants who were part of an OHN, this could be interpreted as indication of a need for OHNs to better align their activities with workforce priorities and to consider and measure perceived value for those activities. Our finding that those who were part of an OHN were almost two times more likely to be involved in the COVID-19 response may indicate greater awareness and access to opportunities through an OHN connection. Further research is needed to fully explore critical areas for intervention and how OHNs can be a vehicle to support a global outbreak response workforce.

In this study, many respondents were affiliated with academic organizations, which may reflect some of the criticism that the One Health concept remains an academic exercise with little practical operationalization (38, 39). However, a substantial proportion of the survey respondents were affiliated with governmental organizations, highlighting channels toward greater operationalization of One Health. These results can inform sampling designs that ensure greater responses from multiple stakeholder groups, including the public sector, development institutions, and non-governmental organizations (NGOs).

The similar proportion of respondents in our study from human or public health and animal health sectors indicated a balance in input from key professional arenas within the global One Health community. A smaller proportion of respondents identified as being from the environmental sector, the third classical pillar of One Health. This may be a shortcoming in the reach of the survey dissemination to those working in environmental and ecosystem health. Indeed, limited representation of the environmental sector is often noticed in One Health initiatives with calls for better engagement (25, 40). Additionally, with growing awareness of the importance of the social drivers of disease, a specific area for public health strengthening is the integration of social science perspectives into One Health (41, 42). Our study sample included a relatively small proportion of respondents from the social science sector. The results of this study can help in planning sampling for future studies and targeted approaches for reaching out to underrepresented fields.

The survey respondents who were involved with the COVID-19 response reported various types of work, geographic level of response, and skills and areas of expertise applied. Further studies are needed to investigate other aspects of worker pandemic response activities, such as the extent of involvement in terms of time used or proportion of working time allocated. Furthermore, knowing the impact of other multisectoral workforce involvement outside of OHNs would be useful to ascertain lessons learned from this pandemic.

The survey respondents reported a number of barriers that hindered involvement in COVID-19 response activities, although the reason for the barriers was beyond the scope of this study and merits further investigation. These results highlight opportunities for regional OHNs, as well as other actors, to find tailored solutions to enable involvement and activation of professional expertise. This might include enhanced dissemination of relevant opportunities, as exemplified by the joint GOARN call to action (28), paths for involvement for experts across the fields, and targeted funding programs for OHN support.

One barrier to involvement in COVID-19 response activities reported was lack of opportunities or paths for involvement, which can be addressed at the local, national, regional, and international levels. There is room for improvement in the extent to which One Health is taught and embraced in professional education across sectors with calls for the structured incorporation of One Health into professional degree programs (24). OHNs are well-positioned to provide the targeted One Health professional development, continuing education, and workforce training needed. The USAID One Health Workforce Next Generation Project, for example, is specifically building the capacity of OHNs worldwide to prevent, detect, and respond to COVID-19 and other infectious diseases (43). A deeper analysis of the barriers and facilitators, matched with strategies to address them, could help to inform local, national, and global initiatives to guide workforce policy and management in light of what we have learned from COVID-19 (37, 44).

This study had some relevant limitations. A major one was bias due to the dissemination routes and snowball sampling method, which oversampled the One Health community and possibly also those involved in COVID-19 response activities. While the geographical distribution of the respondents reflected the global reach of OHN networks, it also revealed shortcomings in the recruiting of survey participants. These results, however, highlight where OHNs need to focus attention for inclusion by, for example, addressing language barriers or other obstacles to participation in the study and by better coordination across OHNs to expand reach and leverage resources.

Collider bias also could have affected our results and limited the ability to make comparisons between those who reported being part of an OHN and those who did not. Other limitations included an English-only questionnaire, self-reporting, and general constraints of questionnaire studies. For example, despite careful design and piloting, some concepts in the questions may have been understood differently by some respondents. Free text responses might have also provided different results for some of the questions beyond the options provided. Importantly, the concept of “OHN” was not defined in this work. Moreover, lack of sociodemographic data was a limitation. Future studies should include variables, such as age, ethnicity, gender, and career stage to evaluate the presence of selection and response bias, as well as any confounding variables. This would also provide insight into any disparities observed based on these sociodemographic variables as a first step to addressing them.

Operationalizing One Health will need to be adapted to build the workforce competencies required for the post-COVID-19 future. Despite the proven benefit of One Health approaches during pandemics (14), the establishment of an effective multisectoral workforce remains problematic. This is due, in part, to the lack of integration of the One Health approach into current international treaties (45). For example, shortcomings in including the One Health approach in the International Health Regulations (IHR) have been linked to delayed and suboptimal action during the early response to COVID-19 (36, 45). Calls for global governance and financing mechanisms to advance One Health as a guiding principle to reform global public health are key to actionable system-level solutions to scale up pandemic preparedness, including workforce development (46). Greater understanding of the activities and needs of the One Health workforce during a pandemic response helps to pave the way for meaningful integration into coordinated and shared strategies for preventing, detecting, and responding to global public health emergencies.

## Data Availability Statement

The coded dataset of de-identified survey responses used in this study is included as Supplementary Material. Further inquiries can be directed to the corresponding author.

## Ethics Statement

The research was exempted from ERC review by the Ad-Hoc Covid-19 Research Ethics Review Committee (WHO ERC/Covid-19). Participation was completely voluntary, no questions were mandatory to answer, and the respondents consented for their answers being used by submitting them. The data were anonymous; the dataset was checked for completeness of anonymity and de-identified. No potentially identifiable human data are presented in this study.

## Author Contributions

LSt, VD, and CS conceptualized the study. LSt and CS verified the underlying data and had access to all data. LSe ran the statistical analyses and created the figures. PJ, JB, LSt, and VD contributed to the data analysis. LSt prepared the first manuscript draft. All authors participated in the design of the study, had access to the de-identified dataset, participated in interpretation of the results, contributed to writing and editing the paper, and approved the final manuscript for publication.

## Funding

This work was supported by the One Health Commission and the European Union Horizon 2020 Research and Innovation Program under grant agreement number 773830: One Health European Joint Programme. The European Union Horizon 2020 Research and Innovation Program provided funds for the open access publication fee. The funders had no role in the study design, data collection, data analysis, data interpretation, or writing the manuscript.

## Conflict of Interest

The authors declare that the research was conducted in the absence of any commercial or financial relationships that could be construed as a potential conflict of interest.

## Publisher's Note

All claims expressed in this article are solely those of the authors and do not necessarily represent those of their affiliated organizations, or those of the publisher, the editors and the reviewers. Any product that may be evaluated in this article, or claim that may be made by its manufacturer, is not guaranteed or endorsed by the publisher.
