# Supplementary material for: Participation in One Health Networks and Involvement in the COVID-19 Pandemic Response: A Global Study
Source: Front Public Health. 2022 Feb 24;10:830893. doi: 10.3389/fpubh.2022.830893 (PMC8907588; doi:10.3389/fpubh.2022.830893)
Supplement: Supplementary file 1 [file Data_Sheet_1.zip › Supplementary Figure 1.pdf]

Supplementary Figure 1. OHN helpfulness toward contribution to COVID-19 response among survey respondents affiliated with OHN

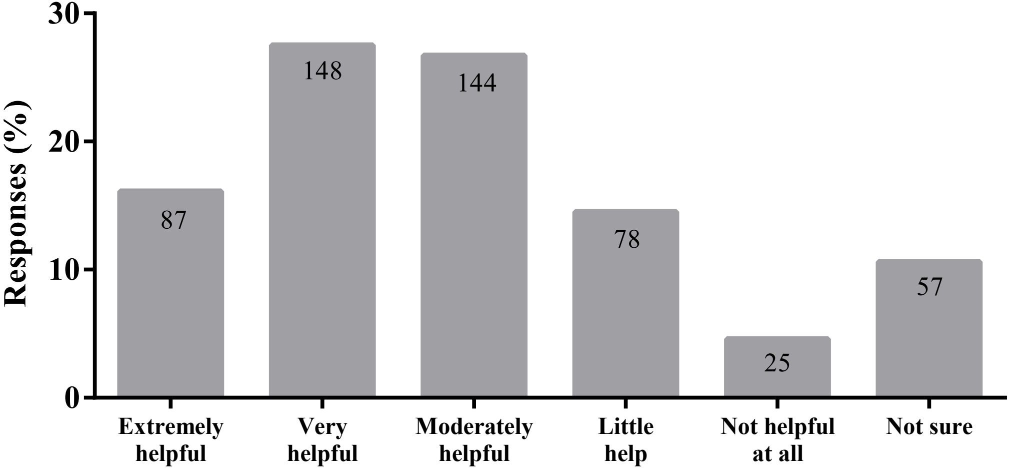

Number of complete answers N=539, no response=61, did not participate in COVID-19 response=188
