# Supplementary material for: Participation in One Health Networks and Involvement in the COVID-19 Pandemic Response: A Global Study
Source: Front Public Health. 2022 Feb 24;10:830893. doi: 10.3389/fpubh.2022.830893 (PMC8907588; doi:10.3389/fpubh.2022.830893)
Supplement: Supplementary file 1 [file Data_Sheet_1.zip › Supplementary Material 1.pdf]

Supplementary Material 1

STROBE Statement—Checklist of items that should be included in reports of ***cross-sectional studies***

	Item No	Recommendation	Section
**Title and abstract**	1	(*a*) Indicate the study's design with a commonly used term in the title or the abstract	Abstract
		(*b*) Provide in the abstract an informative and balanced summary of what was done and what was found	Abstract
**Introduction**			
Background/rationale	2	Explain the scientific background and rationale for the investigation being reported	Introduction
Objectives	3	State specific objectives, including any prespecified hypotheses	Introduction
**Methods**			
Study design	4	Present key elements of study design early in the paper	Abstract, Materials and Methods
Setting	5	Describe the setting, locations, and relevant dates, including periods of recruitment, exposure, follow-up, and data collection	Materials and Methods
Participants	6	(*a*) Give the eligibility criteria, and the sources and methods of selection of participants	Materials and Methods
Variables	7	Clearly define all outcomes, exposures, predictors, potential confounders, and effect modifiers. Give diagnostic criteria, if applicable	Materials and Methods
Data sources/ measurement	8*	For each variable of interest, give sources of data and details of methods of assessment (measurement). Describe comparability of assessment methods if there is more than one group	Materials and Methods
Bias	9	Describe any efforts to address potential sources of bias	Materials and Methods
Study size	10	Explain how the study size was arrived at	Materials and Methods
Quantitative variables	11	Explain how quantitative variables were handled in the analyses. If applicable, describe which groupings were chosen and why	NA
Statistical methods	12	(*a*) Describe all statistical methods, including those used to control for confounding	Materials and Methods
		(*b*) Describe any methods used to examine subgroups and interactions	Materials and Methods
		(*c*) Explain how missing data were addressed	Materials and Methods
		(*d*) If applicable, describe analytical methods taking account of sampling strategy	Materials and Methods
		(*e*) Describe any sensitivity analyses	NA

**Results**			
Participants	13*	(a) Report numbers of individuals at each stage of study—eg numbers potentially eligible, examined for eligibility, confirmed eligible, included in the study, completing follow-up, and analysed	Results
		(b) Give reasons for non-participation at each stage	NA
		(c) Consider use of a flow diagram	NA
Descriptive data	14*	(a) Give characteristics of study participants (eg demographic, clinical, social) and information on exposures and potential confounders	Results, Tables
		(b) Indicate number of participants with missing data for each variable of interest	Results, Tables
Outcome data	15*	Report numbers of outcome events or summary measures	Results
Main results	16	(*a*) Give unadjusted estimates and, if applicable, confounder-adjusted estimates and their precision (e.g., 95% confidence interval). Make clear which confounders were adjusted for and why they were included	Results
		(*b*) Report category boundaries when continuous variables were categorized	NA
		(*c*) If relevant, consider translating estimates of relative risk into absolute risk for a meaningful time period	NA
Other analyses	17	Report other analyses done—e.g., analyses of subgroups and interactions, and sensitivity analyses	Results
**Discussion**			
Key results	18	Summarise key results with reference to study objectives	Discussion
Limitations	19	Discuss limitations of the study, taking into account sources of potential bias or imprecision. Discuss both direction and magnitude of any potential bias	Discussion
Interpretation	20	Give a cautious overall interpretation of results considering objectives, limitations, multiplicity of analyses, results from similar studies, and other relevant evidence	Discussion
Generalisability	21	Discuss the generalisability (external validity) of the study results	Discussion
**Other information**			
Funding	22	Give the source of funding and the role of the funders for the present study and, if applicable, for the original study on which the present article is based	Author Contributions, Funding

*Give information separately for exposed and unexposed groups.

Reference:

von Elm E, Altman DG, Egger M, Pocock SJ, Gøtzsche PC, Vandenbroucke JP, et al. The Strengthening the Reporting of Observational Studies in Epidemiology (STROBE) statement: guidelines for reporting observational studies. Lancet Lond Engl. 2007; 370:1453–7.
