# Supplementary material for: Participation in One Health Networks and Involvement in the COVID-19 Pandemic Response: A Global Study
Source: Front Public Health. 2022 Feb 24;10:830893. doi: 10.3389/fpubh.2022.830893 (PMC8907588; doi:10.3389/fpubh.2022.830893)
Supplement: Supplementary file 1 [file Data_Sheet_1.zip › Supplementary Material 2.pdf]

One Health networks and workforce response to COVID-19

COVID-19 is a One Health issue. The WHO defines One Health as an approach to designing and implementing programmes, policies, legislation, and research in which multiple sectors communicate and work together to achieve better public health outcomes.

One Health networks (OHNs) have formed around the world to serve professionals with common interests in One Health (OH).

This study aims to describe the contributions of the diverse workforce applying OH to the COVID-19 pandemic and the connection of OHNs to workforce response activities.

We seek your participation in this survey, whether or not you work in OH, are part of a OHN, or are currently involved with COVID-19 response and/or research activities.

The estimated time to complete the survey is 10 minutes. Participation is entirely voluntary. By submitting your answers, you consent to the use of this data for the research purposes indicated.

Please complete this online survey based on your personal experiences and perceptions. Responses will not be viewed to represent the official position of your organization. Individual responses will be anonymous and only aggregated data will be presented.

This is a project of the One Health Commission (OHC) and the One Health European Joint Programme (OHEJP), in partnership with the World Health Organization's Global Outbreak and Response Network (WHO-GOARN).

If you have any questions, please contact:
Cheryl Stroud (OHC; cstroud@onehealthcommission.org)
Pikka Jokelainen (OHEJP; PIJO@ssi.dk)
Victor J. Del Rio Vilas (WHO-GOARN; delriov@who.int)

For more information about this study, please see the Participant Information Sheet at:
https://www.onehealthcommission.org/documents/filelibrary/oh_news/71320_OHN_AC81AC6F559D7.pdf

Thank you for your contribution!

1. Where are you currently located?

2. How did you receive the link to this questionnaire? Select all that apply.

- [ ] Directly from One Health network (OHN) mailing list
- [ ] Forwarded to me from someone else
- [ ] OHN website
- [ ] Other website
- [ ] Social media
- [ ] Not sure
- [ ] Other (please specify)

3. What type of organization do you currently work for or are you affiliated with? Select all that apply.

- [ ] Sub-national level government -- working at local, state, or district level
- [ ] National level government -- working at country level
- [ ] International Agency -- *e.g.*, WHO, ECDC, OIE, FAO, World Bank
- [ ] Academic -- Universities and other institutions of higher education
- [ ] Private sector or for-profit businesses
- [ ] Nonprofit organization -- NGO working at the local, national, regional, or international level
- [ ] Individual not working or affiliated with an organization
- [ ] Other (please specify)

4. In what sector do you currently work? Select all that apply.

- [ ] Animal health
- [ ] Human or public health
- [ ] Environmental health
- [ ] Ecosystem health
- [ ] One Health
- [ ] Social sciences
- [ ] Other (please specify)

5. Do you consider yourself part of any One Health network (OHN)?

- ( ) Yes
- ( ) No

6. Please indicate if you have ever participated in these One Health network (OHN) activities. Select all that apply.

- ☐ Attended *in-person* OHN conference/meeting
- ☐ Attended *online* OHN conference/meeting
- ☐ Attended OHN hosted webinar
- ☐ Co-authored OH publication with OHN colleague
- ☐ Followed OHN on social media
- ☐ Invited other professionals to OHN activities
- ☐ Organized OHN activity
- ☐ Participated in integrated OHN project
- ☐ Participated in OHN workgroup/taskforce/committee
- ☐ Participated in OHN offered training
- ☐ Presented on OH topic for OHN
- ☐ Received communications from OHN list
- ☐ Used OHN to disseminate information

7. Please list any other OHN activities you have participated in.

8. Have you been involved with any COVID-19 response activities, including COVID-19 research?

- ○ Yes
- ○ No

9. Which of the following factors have been ***barriers*** to your participation in COVID-19 response and/or research? Select all that apply.

- ☐ There were no barriers to my participation
- ☐ Lack of personal interest
- ☐ Lack of organizational interest
- ☐ Not part of my job
- ☐ No opportunity or path for involvement
- ☐ No time
- ☐ No financial support
- ☐ Don’t know how to get involved
- ☐ Other (please specify)

10. Which of the following factors have ***facilitated*** your participation in COVID-19 response and/or research? Select all that apply.

- [ ] I have not participated in COVID-19 response and/or research
- [ ] Personal interest
- [ ] Organizational interest
- [ ] Part of established duties at my current job
- [ ] Part of a new project/special deployment for COVID-19
- [ ] Availability of new COVID-19 funding
- [ ] I learned of job opportunity through OHN
- [ ] I learned of volunteer opportunity through OHN
- [ ] Other (please specify)

One Health networks and workforce response to COVID-19

11. What is your type of work for the COVID-19 response? Select all that apply.

- [ ] I have not participated in COVID-19 response and/or research activities
- [ ] Research (basic, clinical, operational)
- [ ] Research (COVID-19 diagnostics, treatments, or vaccines)
- [ ] Research (social science, fieldwork)
- [ ] Practice (clinical, public health, laboratory support, data analysis)
- [ ] Health policy and consultation
- [ ] Education (teaching, presentation, training)
- [ ] Writing (blog, commentary, article, other publication)
- [ ] Administration and support
- [ ] Other (please specify)

12. At what level is your COVID-19 response and/or research activities? Select all that apply.

- [ ] I have not participated in COVID-19 response and/or research activities
- [ ] Subnational - local, district, state
- [ ] National - in one country
- [ ] International - in multiple countries

13. What skills/areas of expertise have you applied to the COVID-19 response? Select all that apply.

- ☐ I have not participated in COVID-19 response and/or research activities
- ☐ Animal health
- ☐ Basic research on coronavirus
- ☐ Case management
- ☐ Clinical research
- ☐ Contact tracing
- ☐ Communications and media
- ☐ Community engagement
- ☐ Data management
- ☐ Disease surveillance
- ☐ Environmental health
- ☐ Human clinical care
- ☐ Infection and Prevention Control (IPC)
- ☐ Information/knowledge management
- ☐ Laboratory support and diagnostics
- ☐ Logistics/supply chain
- ☐ Operational research
- ☐ Outbreak or epidemiological research
- ☐ Risk assessment and management
- ☐ Risk communications
- ☐ Social science (e.g., anthropology, sociology, economics)
- ☐ Testing and diagnostics development
- ☐ Vaccine development
- ☐ Other (please specify)

14. To what extent has participating in One Health network (OHN) activities helped you to contribute to the COVID-19 response and/or research?

- ◯ I have not participated in COVID-19 response and/or research activities
- ◯ Extremely helpful
- ◯ Very helpful
- ◯ Moderately helpful
- ◯ Little help
- ◯ Not helpful at all
- ◯ Not sure

15. What OHN offerings do you think are especially useful during the COVID-19 response? Select all that apply.

- ☐ Increased public awareness of the value of OH
- ☐ Information about professional, career, and service opportunities
- ☐ Links to popular media items relevant to OH and current events
- ☐ Networking with professionals across sectors with common interests
- ☐ Opportunities to contribute in ways that my employment does not provide
- ☐ Targeted training opportunities
- ☐ Trusted information about the COVID-19 pandemic
- ☐ Other (please specify)

16. Please share any additional comments you have on One Health networks (OHNs) during the COVID-19 pandemic response.

Thank you for completing this survey!

By submitting your answers you consent to the use of this data for the research purposes indicated.

For more information about this study, please see the Participant Information Sheet at:
https://www.onehealthcommission.org/documents/filelibrary/oh_news/71320_OHN_AC81AC6F559D7.pdf

If you have any questions, please contact :
Cheryl Stroud, OHC, cstroud@onehealthcommission.org
Pikka Jokelainen, OHEJP, PIJO@ssi.dk
Victor del Rio Vilas, WHO-GOARN, delriov@who.int

One Health EJP has received funding from the European Union’s Horizon 2020 research and innovation programme under grant agreement No 773830. The One Health Commission is funded by organizations that support its mission (https://www.onehealthcommission.org/en/sponsorship/).
