# Supplementary material for: Participation in One Health Networks and Involvement in the COVID-19 Pandemic Response: A Global Study
Source: Front Public Health. 2022 Feb 24;10:830893. doi: 10.3389/fpubh.2022.830893 (PMC8907588; doi:10.3389/fpubh.2022.830893)
Supplement: Supplementary file 1 [file Data_Sheet_1.zip › Supplementary Material 3.pdf]

Participant Information Sheet (PIS)

Global Survey: One Health Networks and Workforce Response to COVID-19

This document contains details for the study, including information about data protection and usage of the responses to the questionnaire. You are encouraged to download this document at https://www.onehealthcommission.org/ and store it.

- ***Who is the leader of the work?***
  This work is led by: Dr. Laura Streichert (lcstreichert@gmail.com) and Dr. Cheryl Stroud (cstroud@onehealthcommission.org) from the One Health Commission; Dr. Pikka Jokelainen (PIJO@ssi.dk) and Dr. Ludovico Sepe (ludovico-pasquale.sepe@bfr.bund.de) from the One Health European Joint Programme; Dr. Victor del Rio Vilas (delriov@who.int) from the WHO- Global Outbreak Alert and Response Network; and Dr. John Berezowski (john.berezowski@vetsuisse.unibe.ch) from the University of Bern.

- ***What is this study about?***
  This is a global cross-sectional descriptive questionnaire study. This study aims to describe the contributions of the diverse workforce applying One Health (OH) to the COVID-19 pandemic and the connection of One Health networks (OHNs) to workforce response capacity.

- ***Who is funding this work?***
  The One Health EJP has received funding from the European Union's Horizon 2020 research and innovation programme under grant agreement No 773830. The One Health Commission is funded by organizations that support its mission (https://www.onehealthcommission.org/en/sponsorship/).

- ***What are we asking you to do?***
  We are asking you to complete the online survey based on your personal experiences and perceptions. Responses will not be viewed to represent the official position of your organization. Participation is entirely voluntary. Please complete the survey only once.

- ***Who is being asked to participate?***
  We are distributing the survey globally for a wide convenience sample. We seek your responses, whether or not you work in One Health, are part of a One Health network (OHN), or you are currently involved with COVID-19 response and/or research activities.

- ***What are the benefits of participating?***
  By participating in this survey, you contribute to expanding the understanding of the value and role of a One Health approach and of One Health networks in the current COVID-19 pandemic. The results will inform better management of the One Health workflow in future crises.

- ***What happens if you stop the questionnaire half way through?***
  You can leave this survey at any time. If you exit the survey without pressing the "submit" button, your answers are not recorded. Please note that because the questionnaire is anonymous, once your answers are submitted, they cannot be retracted. By submitting your answers, you consent to the use of this data for research purposes.

- ***What are the possible disadvantages or risks of taking part?***
  There are no direct disadvantages resulting from taking part in this survey. However, by answering some questions you might become more aware of potential barriers that you may have encountered.

- ***How are we ensuring the anonymization?***
  No personal identifiable data nor email or IP addresses are collected. Please do not provide details about yourself, your role, or your organization in your answers. The research group will screen the submitted answers and completely remove any answers with such details before data analysis to protect anonymity.

- ***What happens to the information collected and who has access to the data?***
  No personal data are collected that could identify any of the participants. The raw data will be stored securely by the One Health Commission and only used for the purposes of this research project. The full raw dataset will be accessible to the two researchers from One Health Commission listed on this document. The analysis-data (after confirming the data are anonymous) will be coded and used for statistical analyses within the research group. The analysis-data are moved internationally. The completely coded dataset (no open question answers) will be published with the main articles. All results are reported in a way that individual answers are unidentifiable.

- ***How long do we keep your data?***
  Anonymised data will be stored securely by the research group for maximum 5 years. The completely coded dataset will be included as an appendix with any publications from this work.

- ***With whom do we share your data?***
  The completely coded dataset will be made publicly available for use by the scientific community. The answers to open questions will not be included in this dataset. Data could be further processed for archiving purposes in the public interest, or for historical, scientific or statistical purposes.

- ***What is the research output of this questionnaire?***
  The data collected will be used to develop and publish academic research papers. These will be published in open access journals.

- ***Researcher contact details and contact points if any issues are raised:***
  If you have any questions or concerns about the research, then please contact Dr. Cheryl Stroud (cstroud@onehealthcommission.org), Dr. Pikka Jokelainen (PIJO@ssi.dk) or Dr. Victor del Rio Vilas (delriov@who.int). If ever required, an external independent advisor would be consulted.
